# Noisy Synaptic Conductance: Bug or a Feature?

**DOI:** 10.1016/j.tins.2020.03.009

**Published:** 2020-06

**Authors:** Dmitri A. Rusakov, Leonid P. Savtchenko, Peter E. Latham

**Affiliations:** 1 Queen Square UCL Institute of Neurology, University College London, Queen Square, London, WC1N 3BG, UK; 2 Gatsby Computational Neuroscience Unit, University College London, 25 Howland Street, London, W1T 4JG, UK

**Keywords:** synaptic noise, information transfer, optimal synapse

## Abstract

More often than not, action potentials fail to trigger neurotransmitter release. And even when neurotransmitter is released, the resulting change in synaptic conductance is highly variable. Given the energetic cost of generating and propagating action potentials, and the importance of information transmission across synapses, this seems both wasteful and inefficient. However, synaptic noise arising from variable transmission can improve, in certain restricted conditions, information transmission. Under broader conditions, it can improve information transmission per release, a quantity that is relevant given the energetic constraints on computing in the brain. Here we discuss the role, both positive and negative, synaptic noise plays in information transmission and computation in the brain.

## Highlights

Synaptic transmission is noisy, partially because neurotransmitter release is unreliable but also because synaptic receptor current varies stochastically.
When synaptic input is below the cell spike threshold, adding noise may push it above the threshold, thus improving information transmission.
Synaptic noise can widen the dynamic range of transmission.
Synaptic noise allows synapses to communicate their degree of uncertainty.
Noise is a ubiquitous feature of brain activity and is likely to be a critical element in any theory of how the brain works.

## Neuronal Communication is Noisy

Neurons communicate primarily through chemical synapses, and that communication is critical for proper brain function. However, chemical synaptic transmission appears unreliable: for most synapses, when an action potential arrives at an axon terminal, about half the time, no neurotransmitter is released and so no communication happens. Even strong and reliable synapses, such as the neuromuscular junction or the calyx of Held, operate by using multiple unreliable release sites [1]. Furthermore, when neurotransmitter is released at an individual synaptic release site, the size of the local postsynaptic membrane conductance change is also variable. Given the importance of synapses, the energetic cost of generating action potentials, and the evolutionary timescales over which the brain has been optimized, the high level of synaptic noise seems surprising. Here we ask: how surprised should we be? More to the point: is synaptic noise a bug, or is it a feature? Whilst the role of unreliable release on neural network performance has been explored in multiple studies (see [2., 3., 4.] for informative reviews), here we expand on these views, in particular giving consideration to noisy **synaptic conductance** (see Glossary).

## Noise in Nonlinear Communication Channels

**Figure 1:**
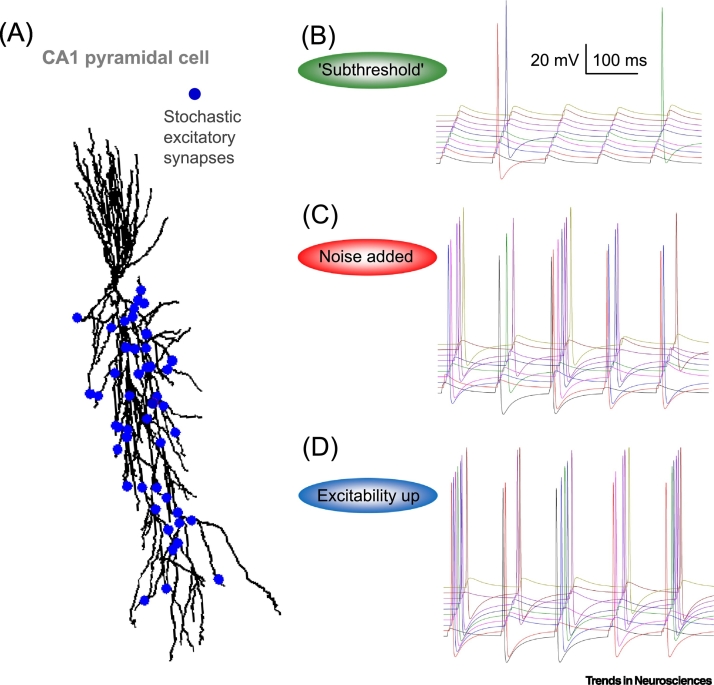
Increasing Synaptic Input Noise or Postsynaptic Cell Excitability Can Improve Signal Transfer: An Illustrative Example. (A) A basic multisynaptic neuron model: reconstructed CA1 pyramidal cell model equipped with known membrane mechanisms [57] (ModelDB accession number 2796, NEURON database). Fifty excitatory inputs (random scatter, blue dots) generate biexponential conductance change (rise and decay time, 0.1 ms and 1.2 ms, respectively), with the same onset but stochastically, in accord with their release probability, *P*_*r*_; simulations with NEURON 7.2 [58], variable time step *dt*, t = 34°C. (B) Somatic voltage traces in response to synchronous activation of 50 unreliable synapses (*P*_r_ = 0.5), five stimuli at 10 Hz, no noise (conductance coefficient of variation, CV = 0), 'near-subthreshold' activation (peak synaptic conductance *G*_s_ = 7.6 pS; somatic sodium channel conductance *G*_Na_ = 0.032 mS/cm^2^). (C) As in (B) but with synaptic conductance noise (CV = 0.05) leading to increased spiking. (D) As in (B) but with cell excitability increased (somatic sodium channel conductance doubled to *G*_Na_ = 0.064 mS/cm^2^). A fully functional model example and illustrative movie files can be downloaded from http://www.sciencebox.org/Neuroalgebra/TINSWeb/

When communicating information, noise would seem to be universally bad. Static on the radio, background noise during a conversation, grainy videos – they all make it harder to extract interesting information. However, for **nonlinear systems** – especially when a threshold is involved, as in spike generation – noise can be helpful [5., 6., 7.]. Most notably, if a noise-free presynaptic signal is too small, it will never generate a spike and thus will not transmit any information. Add a small amount of noise, and, at least sometimes, a spike will occur. Thus, a subthreshold sensory signal has a better chance of being detected when noise is added [8,9]. This phenomenon is, very broadly, known as **stochastic resonance** [3,10,11], and it is observable in electrophysiological experiments: add a small amount of noise to **subthreshold synaptic currents**, and the probability of a spike can go way up [12]. A simple multisynaptic neuron model (Figure 1A) illustrates this point. With no noise in the synaptic conductance, there are very few spikes (Figure 1B), but adding a small amount of variability in the postsynaptic conductance can produce fairly reliable spikes (Figure 1C). Note, however, that decreasing threshold for the generation of an action potential can produce even more reliable spikes (Figure 1D), which has a higher somatic sodium conductance, making the cell more excitable compared with Figure 1B. Although in a wider context we cannot equate more spiking with more information, we will, in the interest of simplicity, assume that is the case here.

Abstractly, the ability to communicate information depends on three things: the range of possible input signals, the noise around those signals, and the transformation from input to output (Box 1 provides a commonly accepted definition of information). For neurons, the transformation from input to output yields either a spike or no spike. In that case, communication is optimal when the integrated input to a neuron is either well above or well below threshold relative to the noise. In such a regime, a spike provides unambiguous information about the input: it tells downstream neurons whether the input was high or low. But typically, the signal and noise cannot be adjusted independently; in particular, the **synaptic drive** to a neuron is not **bimodal** – it is graded. So, the simple, optimal, regime is not realized. Consequently, the question of whether synaptic noise is a bug or a feature depends on how the signal and noise interact. For instance, if increasing noise made synaptic drive more bimodal, it could, at least in principle, increase information transmission. And we have already seen that adding noise can increase the reliability of output spikes (Figure 1C). But we have also seen that increasing cell excitability, and thus lowering the threshold for a spike, can do the same thing (Figure 1D). Which one is better, and when?

Box 1 **Defining (Mutual) Information**

Spike trains convey information about presynaptic input. Intuitively, this means that if you were to observe spikes from a neuron, you would know more about its synaptic drive than if you did not observe spikes. To quantify this intuition, we need a way to measure knowledge – or, as is much more common, uncertainty. There are many ways to do this, but an especially convenient one is entropy, which is a relatively direct measure of uncertainty; the higher the entropy of a distribution over some variable (i.e., the broader the distribution), the more the uncertainty about that variable (according to Equations (I), (II) as follows). With this choice, the difference in entropy is the Shannon mutual information [59], which indicates how much information about the input signal we can glean from the output signal (Figure I).

The entropy of a continuous distribution *P*(*x*), often denoted *H*(*x*), is

(I) $$H\left(x\right)=-\int \mathrm{dxP}\left(x\right)\log_{2}P\left(x\right).$$

If *P*(*x*) is discrete there is an analogous definition; the only difference is that the integral becomes a sum,

(II) $$H\left(x\right)=-\sum_{i}P\left(x_{i}\right)\log_{2}P\left(x_{i}\right).$$

To compute mutual information, we need to compare the entropy before and after some observation (which for us is a spike train). Let’s use *s* for the observation, and P(*x* | *s*) for the distribution of *x* (which for us is the synaptic drive) after observing a spike train. In that case, the conditional entropy, denoted *H*(*x* | *s*) – the average entropy after observing a spike train – is

(III) $$H\left(x|s\right)=-\int \mathrm{dxdsP}\left(s\right)P\left(x|s\right)\log_{2}P\left(x|s\right),$$

with integrals replaced by sums for discrete distributions. The mutual information, *I*(*x*,*s*), between *x* and *s* is the difference between the two entropies,

(IV) $$I\left(x,s\right)=H\left(x\right)-H\left(x|s\right).$$

This definition is sufficiently flexible that *x* could be continuous and *s* discrete, or vice versa. As is not hard to show, information is symmetric; it can also be written

(V) $$I\left(x,s\right)=H\left(s\right)-H\left(s|x\right).$$

Symmetry is important because it tells us that the higher the entropy of a spike train (which typically translates to higher firing rate), the larger the potential for high information. Importantly, though, increasing the firing rate is not guaranteed to increase information, as that may also increase the second term.

**Figure I:**
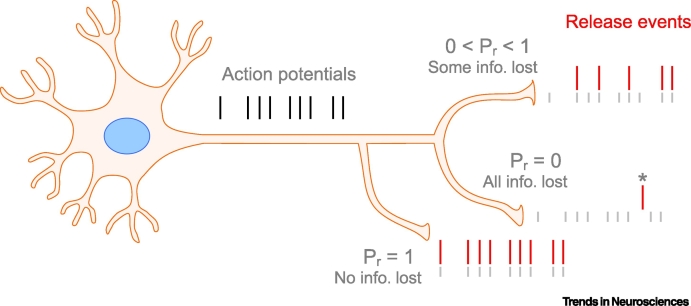
Schematic Illustrating the Mutual Information between a Series of Axonal Spikes and the Corresponding Series of Neurotransmitter Release. This schematic illustrates the mutual information, *I*, between a series of axonal spikes (black bars; indicated with grey on the postsynaptic side) and the corresponding series of neurotransmitter release (red bars). Note: this is different from the mutual information between the input and output because release does not guarantee an output spike. Information depends on the release probability, *P*_*r*_, at each presynaptic site. The asterisk indicates spontaneous (action potential-independent) release.

Alt-text: Box 1

## Sources of Synaptic Noise

To investigate how synaptic noise affects information transmission in realistic situations, we need to understand the sources of noise. There are two main sources. The first, and best studied, are release failures: when an action potential invades the presynaptic terminal, neurotransmitter may or may not be released [13,14]. The average **release probability**, *P*_*r*_ – calculated as one minus the probability of failure – ranges from 0.2 to 0.8 at central synapses [15., 16., 17., 18.], with experiments *in vivo* putting this towards the low end [19]. This generates considerable noise: if ten action potentials arrive at a cell approximately simultaneously, typically somewhere between two and eight of them will cause a release of neurotransmitter, and on any one trial, anywhere from zero to ten will cause neurotransmitter release.

The second source of noise is variability in the synaptic conductance (producing variability in the peak synaptic receptor current) in response to release of one neurotransmitter-filled synaptic vesicle. Fluctuations in the synaptic conductance depend on two main factors. First, synaptic vesicle content can vary depending on prior activity [20,21]. Second, synaptic receptor binding and activation is a **stochastic** process controlled by conformational changes of receptor proteins [22,23]. The lateral mobility of synaptic receptors [24,25] and, possibly, neurotransmitter release sites [26,27], have also been considered a factor. However, recent super-resolution studies have revealed a structural alignment of presynaptic release sites and postsynaptic receptor clusters [28], which should minimise this source of noise, at least on time scale of minutes [29]. Nonetheless, excitatory postsynaptic currents recorded at individual synapses in response to synaptic vesicle release, in basal conditions, vary significantly. A recent study suggested that the architecture of common excitatory synapses is such that it maximises synaptic conductance variability [30]. If so, it would appear that individual excitatory synaptic connections 'prefer' to be noisy.

An additional source of noise affecting synaptic circuit signalling is stochastic fluctuations of the cell membrane potential. Its multiple origins and its effect on performance have recently been reviewed in detail [31] and are outside the scope of this present opinion article.

## How Does Synaptic Noise Affect Information Transmission?

Figure 1 shows that if the spike threshold is set too high, noise from variability in the conductance can improve information transmission. This is not true of release failures: lowering the probability of release decreases the drive to a postsynaptic cell. However, if we conserve transmitter expenditure, by increasing the conductance as we lower the release probability so that the product of conductance and *P*_*r*_ stays constant, the story changes. In this regime, the mean is independent of the release probability and the variance scales as (1– *P*_*r*_)/*P*_*r*_. The variance thus increases as *P*_*r*_ decreases – exactly what we need to drive a cell when the threshold is set too high. Consequently, both failures and conductance variability could be beneficial for information transfer.

**Figure 2:**
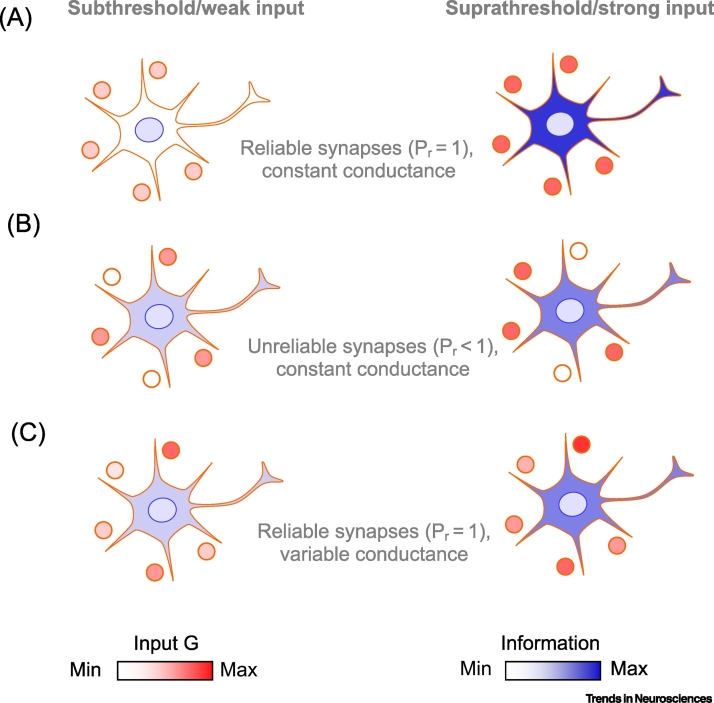
Noisy Synapses Can Be Informationally Advantageous towards the Lower End of the Transmission Dynamic Range. Synaptic inputs are shown by circles, with the intensity of red reflecting their activity. The amount of spiking activity is indicated by the shade of blue, with higher probability of spiking (darker blue) corresponding to higher information rates. (A) Reliable synapses (*P*_r_ = 1) and no variability in postsynaptic conductance. If the threshold is too high there are no spikes (left), but if the threshold is lowered slightly, spike transmission is 100% reliable (right). (B) Unreliable synapses (*P*_r_ < 1) and no variability in postsynaptic conductance, but conductance is proportional to 1/*P*_r_ (to conserve the transmitter expenditure). In this case, postsynaptic spiking is possible under weak input, thus transmitting some information (left). However, under strong input, unreliable synapses lose information [right; compare with (A)]. (C) Reliable synapses (*P*_r_ = 1), but with variability in postsynaptic conductance. Information transmission is about the same as with unreliable synapses with variability in postsynaptic conductance [compare with (B)].

This is illustrated in Figure 2. Figure 2A shows that reliable synapses (*P*_*r*_ = 1) with constant conductance are perfectly efficient, but only when the overall input is above the threshold (compare left and right). In comparison, unreliable synapses (*P*_*r*_ < 1) with conductance scaled to conserve transmitter expenditure (as described earlier) can transfer more information when below threshold, but their performance is worse above threshold (Figure 2B). Not surprisingly, reliable synapses with variability in conductance will behave in a similar way to unreliable synapses with constant conductance (Figure 2C).

The picture that emerges from this analysis is that when the threshold is set too high, noise increases information transmission; otherwise, noise decreases information transmission (see Figure I in Box 2). This suggests that the optimal strategy is to reduce noise as much as possible, then set the threshold for spike generation to maximize information transmission. As we have seen, increasing release probability and reducing conductance variability both reduce noise. In addition, the nervous system has another option: increase the redundancy of synaptic connections – by allowing individual axons to make multiple synaptic connections on a postsynaptic cell [32]. This averages the noise and so partially compensates for synaptic unreliability [33,34]. This is, in fact, the strategy taken by reliable synapses, such as the neuromuscular junction or the calyx of Held, which are equipped with multiple unreliable release sites [1].

Box 2 **Computing (Mutual) Information**

To gain an understanding of how mutual information depends on parameters, consider a simplified scenario in which there is a single presynaptic neuron that either does or does not fire, the output spikes tell us whether or not it fired, time is divided into small bins, say 10 ms, and there are either zero spikes or one spike in each bin.

In this scenario, it is not hard to show that the mutual information (Box 1) is given by

(I) $$I\left(x,s\right)=h\left(\mathrm{qp}\right)-\mathrm{ph}\left(q\right).$$

where *p* is the probability that the presynaptic neuron fires, *q* denotes the probability that the postsynaptic neuron emits a spike given that the presynaptic neuron fired, and *h*(*z*) is the entropy of a Bernoulli random variable with probability *z*,

(II) $$h\left(z\right)=-z\log_{2}z-\left(1-z\right)\log_{2}\left(1-z\right).$$

Assume the postsynaptic neuron fires when its input exceeds a threshold, *θ*, and that the input drive is Gaussian with mean *μ* and standard deviation *σ*. In that case, *q* is a cumulative normal function of (*μ* – *θ*)/*σ*. A plot of mutual information versus *μ* is shown in Figure I for various values of the noise (the thicker the line, the larger the noise). If the mean synaptic drive is above threshold, noise hurts, because it decreases the probability that a presynaptic spike will cause a postsynaptic one; if it is below threshold, noise helps, because it increases the probability that a presynaptic spike will cause a postsynaptic one. In addition, the higher the firing rate of the presynaptic neuron, the higher the mutual information (compare red and blue curves).

These observations suggest that mutual information is maximized when the input is bimodal [either well above or well below threshold, so that *q* = 1 according to Equation II, which means *h*(*q*) is zero], the noise is small, and the output firing rate is large. However, it is unlikely that these are achievable. For instance, presynaptic spikes are spread out over time. Consequently, the synaptic drive is a continuous function of time, and neurons spike as soon as the drive crosses threshold. Thus, synaptic drive is rarely bimodal. In addition, there are energetic constraints on firing rate, so it can’t be arbitrarily large. Note, though, that in the brain, the input information is much larger than the output information – about 1000 times larger, since each neuron receives about 1000 inputs. Consequently, the information lost due to noise added by failures and variability in the size of postsynaptic currents is likely to be small compared with the information that is thrown away by the finite capacity of neurons, an argument made by Levy and Baxter [37].

**Figure I:**
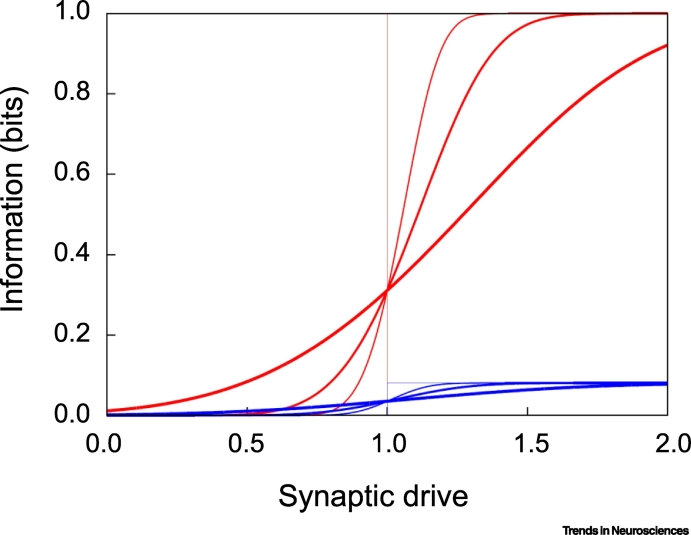
Information (in Bits) Versus Synaptic Drive, *μ*. Red: the probability of a presynaptic spike, *p*, is 0.5, corresponding to an effective presynaptic firing rate of 50 Hz, assuming a time bin of 10 ms. Blue: the probability of a presynaptic spike is 0.01, corresponding to an effective presynaptic firing rate of 1 Hz. Noise levels, *σ*, are 0.0, 0.1, 0.2, and 0.5 (thin to thick lines). The threshold, *θ*, was set to 1.0.

Alt-text: Box 2

Taken to its extreme, if neurons can optimise their threshold, zero noise should always be optimal. However, the continual learning that goes on in the brain involves constant adjustment of synaptic strengths and cell excitability. This should make threshold optimisation a continuously evolving process – one that can never be perfectly realised. Indeed, the large range of timescales over which changes in synaptic strength and in cell excitability occur [35,36] suggest that brain circuits cannot rely on permanently optimised thresholds. Thus, it may be advantageous to use synaptic noise to ensure that some spikes are transmitted rather than relying on an optimal threshold. In this context, release probability is not strictly noise: its use-dependent changes may represent a certain 'informative' operation that contributes to the transmitted message.

So far, we have defined optimality purely with respect to information transmission. However, even if a circuit is optimal from an information transmission standpoint, it is unlikely to be optimal once the cost of neurotransmitter release is considered. There is a relatively simple reason for that: spikes have limited bandwidth (sustained firing rates are almost always well below 100 Hz, with only occasional bursts of a few spikes at 1 kHz), so substantial input information, which occurs when release probability is high and/or input activity is intense, is largely wasted. As shown previously [37], for reasonable output firing rates, release probabilities that maximize information per release are in the range 0.2–0.8; exactly what is seen *in vivo*. Subsequent studies taking into account short-term plasticity [38., 39., 40.] found the same thing: information per release peaked at release probabilities below one. It is easy to see why, at least in the extreme case: if two consecutive presynaptic spikes occur with a small interspike interval, it is more energy efficient to release neurotransmitter on only one of the spikes but double the conductance change than to release neurotransmitter on both spikes without doubling the conductance change. This has the added benefit of increasing slightly the peak conductance, and thus increasing the probability of a postsynaptic spike.

The studies mentioned earlier, on optimal information per release, considered noise only from synaptic failures. However, the same lessons should apply to variability in synaptic conductance but with an important difference: because conductance variability does not affect release probability, if the threshold is set perfectly, adding conductance variability would not increase information per release, and so would seem to be uniformly bad for information transmission per release. However, the fact that excitatory synapses appear to maximise synaptic conductance variability [30] points to the possibility that theory is missing an important role for this kind of noise.

It is also worth mentioning that noise can affect the input–output function of neurons. Modern theories of recurrent spiking networks suggest that most of the time the mean drive to a neuron is subthreshold, and it is temporal fluctuations around the mean that drive activity [31,41., 42., 43., 44.]. Whenever this is the case, increasing the (zero mean) noise – no matter how it is done – increases the probability that a postsynaptic cell will fire. Thus, noise, due either to synaptic failures or variability in peak synaptic conductance, is an energetically cheap way to increase the gain of a neuron (albeit at the cost of larger fluctuations). Noise can also decrease the probability of firing; that happens when the input to a neuron is superthreshold. Thus, the overall effect of noise is to flatten the gain curve (input current to firing rate), and so widen a neuron’s dynamic range. This is something that threshold adjustments cannot do.

## A Computational Role for Noise

It is reasonable to assume that information transmission is the main function of synapses. However, there is another potential role, one rooted in the Bayesian approach to neuroscience [45., 46., 47., 48.]. It has become increasingly clear over the last several decades that organisms – from fly larvae to humans – compute and manipulate probabilities [48., 49., 50., 51.]. This is not surprising; it is impossible to make a good decision of any kind without some measure of uncertainty. For instance, every time we cross the street we have to estimate whether the oncoming car will hit us; because it is impossible to get an exact estimate, we must evaluate how uncertain we are about a collision. Without some estimate of uncertainty, we would not know whether we can cross the street reasonably safely. Much of the work in this area has focused on uncertainty in our perception of the outside world. However, there is another source of uncertainty: the computations the brain performs depend on synaptic strengths, but the correct strengths are not known with certainty. In principle, that degree of uncertainty should be communicated to the rest of the brain. Recently it was proposed that synapses compute their uncertainty and then communicate that uncertainty by adding noise to synaptic weights [52]. With this theory, variability in synaptic strength – caused by failures, variability in conductance size, or both – is a feature, not a bug; it is used by the rest of the brain to estimate uncertainty, and, ultimately, to determine confidence. Whether or not synapses do this is currently not known, but it is an avenue worth exploring, we would argue, and one that raises concrete questions for future research.

In our discussion of information transmission, we have operated under the assumption that noise is useful only if the spike threshold is too low. However, noise can be useful in and of itself. For instance, it can boost behavioural variability – an essential feature in most, if not all, adversarial games. It is also useful for exploratory behaviour, one prominent example being identifiable random number generators in birdsongs [53]. In some instances, however, synaptic noise could be unimportant, simply forced on the brain by biological reality. And the cost of noisy synapses may not be very high: chaotic dynamics [54] already introduces a large amount of noise in neural circuits, so adding a little more probably does not cost much in terms of computational capabilities. For instance, the noise associated with failures should have very little effect on the accuracy of computations [55], and the approximations that the brain makes have a much larger effect on performance than the noise found in either synapses or chaotic dynamics [56].

## Concluding Remarks

Two key physiological phenomena contribute to what we consider synaptic noise: stochastic neurotransmitter release and variability in postsynaptic conductance given release. Multiple lines of experimental and theoretical evidence suggest that, at least in certain conditions, synaptic noise improves information handling by brain circuits. Here we attempted to discuss, and illustrate, how and when this is the case. Although much progress has been made, we still lack a deep understanding of the role of noise in the nervous system. In particular, it is currently unknown how noise interacts with plasticity at the synaptic level, learning at the behavioural level, or computations at the circuit level. These are all fundamental issues and represent fertile ground for experimental and theoretical exploration. No matter what its role, noise is a ubiquitous feature of brain activity and is likely to be a critical element in any theory of how the brain works (see Outstanding Questions).

Outstanding Questions What is the experimental relationship between synaptic noise and synaptic morphology?

How stable are conductance fluctuations at individual synapses over short periods of time?

Is there a relationship between synaptic current variability and a synapse’s position on the dendritic tree?

How does synaptic noise influence dendritic signal integration?

Do central synapses change their 'noisiness' with development and aging?

Are there subgroups of synapses or cells with distinct magnitudes of conductance variability?

Are there synaptic circuits or brain regions with distinct magnitudes of noise?

Does the magnitude of synaptic noise correlate with a neural circuit’s ability to undergo plastic changes?

Does the magnitude of neural network noise correlate with the animal's ability to learn?

Alt-text: Outstanding Questions

## Glossary

**Bimodal**: having two peaks in the distribution, so that typical values are either, say, high or low.
**Nonlinear system**: a system in which the relationship between input and output is not linear.
**Release probability**: average probability with which a synaptic vesicle is released upon the arrival of an action potential.
**Stochastic**: probabilistic; containing an element of uncertainty.
**Stochastic resonance**: a phenomenon in which adding noise increases the probability that a signal will reach threshold for detection.
**Subthreshold synaptic currents**: synaptic receptor-generated membrane current that is too weak to a neuron to fire.
**Synaptic conductance**: peak cell membrane conductance arising through the opening of postsynaptic receptor channels.
**Synaptic drive**: integrated effect of all active synaptic input to a neuron.
